# Leveraging User Experience to Improve Video Consultations in a Cardiology Practice During the COVID-19 Pandemic: Initial Insights

**DOI:** 10.2196/19771

**Published:** 2020-06-25

**Authors:** Pieter Vandekerckhove, Yves Vandekerckhove, Rene Tavernier, Kelly De Jaegher, Marleen de Mul

**Affiliations:** 1 Erasmus School of Health Policy and Management Erasmus University Rotterdam Netherlands; 2 Department of Cardiology AZ Sint-Jan Brugge Brugge Belgium

**Keywords:** telemedicine, design thinking, cardiology, patient, COVID-19, user experience

## Abstract

During the coronavirus disease (COVID-19) pandemic, cardiologists have attempted to minimize risks to their patients by using telehealth to provide continuing care. Rapid implementation of video consultations in outpatient clinics for patients with heart disease can be challenging. We employed a design thinking tool called a customer journey to explore challenges and opportunities when using video communication software in the cardiology department of a regional hospital. Interviews were conducted with 5 patients with implanted devices, a nurse, an information technology manager and two cardiologists. Three lessons were identified based on these challenges and opportunities. Attention should be given to the ease of use of the technology, the meeting features, and the establishment of the connection between the cardiologist and the patient. Further, facilitating the role of an assistant (or virtual assistant) with the video consultation software who can manage the telehealth process may improve the success of video consultations. Employing design thinking to implement video consultations in cardiology and to further implement telehealth is crucial to build a resilient health care system that can address urgent needs beyond the COVID-19 pandemic.

## Introduction

The coronavirus disease (COVID-19) crisis has challenged health care professionals to rapidly reduce face-to-face consultations. To ensure care continuity, the use of telehealth is recommended [1]. Telehealth refers to the use of electronic services to support a broad range of remote services, such as patient care, education, and monitoring [2]. Many health systems have already invested in telehealth, and some primary care practices in the United States have appeared to adopt telehealth almost instantly [3-5].

In Belgium, cardiologists remotely triaged patients who were originally scheduled for face-to-face consultations; however, many consultations were postponed during the lockdown period. Some patients are afraid to come to the hospital due to the risk of contracting COVID-19. This increases the likelihood that patients will stay at home or postpone consultations despite deteriorating symptoms. In fact, a declining incidence of acute myocardial infarction has been witnessed in the United States during the COVID-19 pandemic [6]. However, early detection of atrial fibrillation is crucial to prevent stroke, which is a leading cause of death globally [7,8]. In addition, early detection of heart failure is necessary to prevent hospitalization and death [9].

Telehealth can help mitigate these risks by enabling continued monitoring of patients. Various telehealth solutions can be leveraged for remote cardiology monitoring [1], such as video communication software and implanted devices. The adoption of these tools is now being facilitated because financial and reimbursement restrictions are being lifted; however, further measures are needed for wider adoption of telehealth [4,10,11].

Given that rapid acceptance of telehealth during COVID-19 is critical, telehealth technologies must be easy to implement and to scale up. To achieve this, we employed design thinking [12,13] to learn from the experiences of a cardiology practice in a regional Belgian hospital where video consultations are rapidly being implemented.

## Design Thinking

Design thinking aims to identify and solve problems in a systematic and collaborative way [12,13]. Collaborative design methods are widely used to improve electronic health (eHealth) [14], including the development of eHealth to assist heart patients with self-management [15-19]. However, design thinking research focusing on video communication software with heart patients is lacking. We used a design thinking tool called a customer journey to empathize with all stakeholders, identify the challenges facing each stakeholder, and identify opportunities to redesign the service [12]. To develop the customer journey, PV conducted telephone interviews to map the experience of the lead hospital information technology (IT) manager (who liaised with the legal team), a research nurse (KDJ), the treating cardiologist (YV), and the head of the Department of Cardiology (RT). Five patients with an implanted cardioverter-defibrillator device (ICD) were interviewed by a nurse (KDJ) shortly after the end of each video consultation about their experience. The data were analyzed in PowerPoint (Microsoft Corporation).

The treating cardiologist (YV) identified 13 patients with an ICD who were scheduled for face-to-face consultations in the outpatient clinic. The ICD patient population was prioritized because these patients could especially benefit from telehealth due to the opportunity to access their heart monitoring data remotely. These patients did not have any urgent needs, did not require a physical examination with hospital equipment, and had a telephone number on record. Of these 13 patients, 5 (38%, aged 43-64 years) were eligible for a video consultation. Of the 8 patients who were not eligible, 2 (25%) lacked a smartphone or computer, 2 (25%) were not reachable by telephone, 3 (38%) lacked the functionality for remote monitoring, and 1 (13%) had progressed to needing more urgent care.

## Challenges and Opportunities for Stakeholders

**Figure 1:**
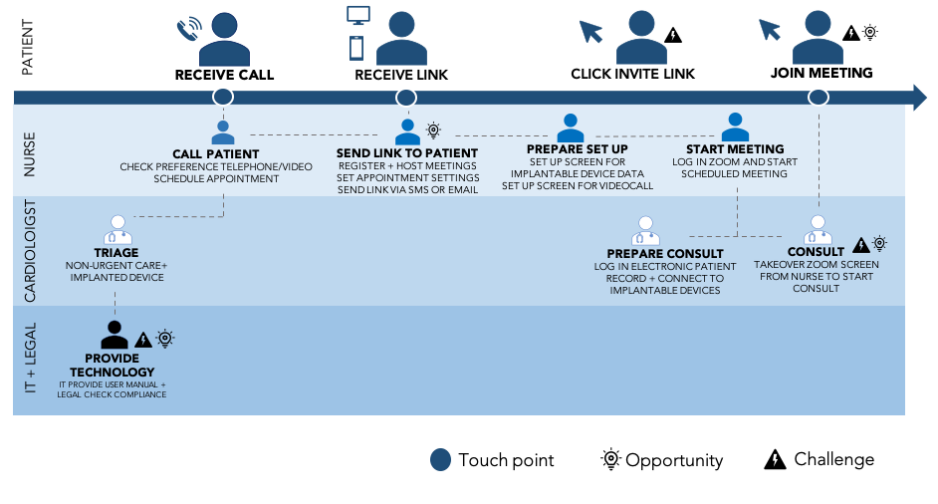
Customer journey of a video consultation for a patient with an implanted device. IT: information technology. SMS: short message service.

The customer journey (Figure 1) shows the actions taken by stakeholders in parallel rows and the touch points (blue and white circles) where the patient is in contact with health care professionals. Three areas of challenges (triangles) and opportunities (lightbulbs) are illustrated: the lower left area is related to the provision of technology, the center area is related to inviting the patient to the consultation, and the right area relates to when the patient joins the meeting and the video consultation starts.

### Video Consultation Technology: Need for Integration, Fewer Steps, and Long-Term Prospects

Available video consultation solutions were explored with the IT and legal teams based on ease of use and suitability for the patient group, short-term implementation for all stakeholders, adaptability, financial constraints, and compliance with the European Union General Data Protection Regulation (GDPR). Skype for Business (Microsoft Corporation) was accessible by all physicians, as it was installed across the hospital on desktop computers. Given its strong security, lack of additional cost, and availability in the hospital, this appeared to be a good option. However, Skype for Business is a rigid software package that will soon be replaced by Microsoft Teams. Eventually, video communication software will be integrated into the electronic patient record software, as is already the case in larger academic hospitals in Belgium. Therefore, Skype for Business demonstrated little adaptability and no long-term prospects. Given that it would still be necessary to provide training to health care professionals and patients, other software was considered. No solution could be identified that integrated video communication software with ICD monitoring, which would suit the patients best (challenge). However, other video communication software was tested, including Zoom (Basic account, Zoom Video Communications Inc). The cardiologist was given remote training on Zoom by the researchers while they simultaneously tested its functionalities compared to Skype for Business. They considered that Zoom was easier to use, as the patient would not need to install a software package on their computer and would need to complete fewer steps to use the desktop version or smartphone app (opportunity). The IT and legal teams had heavy workloads and were only able to provide limited technical support, such as a user manual.

### Receiving the Meeting Invitation: Adapting the Settings

The nurse first called each patient to make an appointment for a consultation by telephone or video. If the patient preferred a video call, they were asked if they would like to receive the call through an app on their smartphone or on a desktop computer. Video consultation appointments were made using Zoom. To reduce the workload for the cardiologist, the nurse was responsible for making appointments and was then made the host of each meeting (opportunity). Here, opportunities were identified to improve safety by changing meeting settings with a password. Other settings were changed to reduce the number of steps the patient was required to take; immediate activation of the camera was enabled when joining a meeting, bypassing the waiting room function. After these steps were completed, a link with the invitation to the video consultation was sent to the patient. Here, another opportunity was identified to circumvent email use if the patient planned to use the app by sending them the link via SMS text message. However, this required the nurse to use an anonymous mobile phone number. Patients using the desktop version of Zoom were sent an invitation link via email.

### Joining Meetings: Need for a Virtual Assistant

Before the start of a video consultation, the nurse prepared two screens (opportunity). On one screen, the cardiologist could log in to the patient’s device, while the other screen was prepared to show the video communication software. For all the video consultations, a backup plan was established to switch to telephone and continue the consultation on an audio-only basis. The nurse was present throughout the entire VC process to help manage problems with communication connections and record the experiences of the patient and the cardiologist.

In 2 of the 5 video consultations, the patient and the cardiologist were connected with both audio and video via Zoom. In the four cases in which a video connection was established, even without audio (replaced by audio from a telephone), the patients were positive about the experience; however, for the one case in which the video connection could not be established, the patient was disappointed. Key challenges were identified due to failures of the video or audio on either the patient’s or the cardiologist’s side (3 times): in one case, the cardiologist’s computer ran too slowly, in another case, the patient had not signed into the Zoom meeting, and in another case, the patient could not join the Zoom meeting. In addition, the patients were not always immediately in front of their devices at the start of the meeting. The nurse had to attempt to manage these problems in the moment on both the cardiologist and patient sides. However, connections could not always be established or re-established. This reveals the need for more advanced training on the use of video communication software before adopting video consultations, for both patients and health care professionals.

Opportunities were identified to improve the audio and video connections between the patient and the cardiologist. First, the nurse could test the Zoom video and audio links with the cardiologist’s and patient’s devices before the start of the consultation. This would require the nurse to be the host for all scheduled video consultations before the consultations start. For example, before the first consultation started, the nurse as the host could conduct a test conversation using the cardiologist’s device to determine if the audio and video were functional and if the connection with the heart device was functioning. Following this, the cardiologist could take over the account from the nurse to start the consultation. The nurse could then prepare the next scheduled patient for their video consultation by starting the next meeting as the host and checking the audio and video connections between the patient’s device and the local device by holding a test conversation.

Currently, there are technical challenges in implementing this workflow, as the meeting host cannot simultaneously start and manage multiple meetings [20]. No other video communication software could be immediately identified that would overcome this obstacle. It may be possible for a chatbot or similar automated diagnostic system to help the patient navigate the steps on their own to test the audio or video connection. All these potential solutions would require additional training for the patients and health care professionals.

## Lessons to Improve Video Consultations in Cardiology

We employed a design thinking tool called a customer journey, which revealed several challenges and opportunities for stakeholders in a cardiology practice when testing video consultation software. Three lessons were identified to improve the experience for stakeholders:

### Ease of Using the Technology

Attempt to reduce the number of clicks or screens that must be navigated to get to a meeting and preferably avoid downloading or registration of software and activation of a microphone or video camera (these appear to be easier to manage on a smartphone). Ideally, provide the ability to access data on implanted devices and video communication software in one integrated software solution.

### Meeting Features

Ensure the video consultations are secure (ie, use a password and data encryption in line with the GDPR) and facilitate the establishment of video and audio connections by automatically starting microphone and video devices for both the health care professional and the patient. Use a convenient method to send information about the meeting to the patient, such as an SMS text message or email, depending on the wishes of the patient.

### Management of Video and Audio Connections

Reduce the time spent preparing and managing connectivity. One option would be for a nurse to concurrently host multiple meetings to streamline the process of switching between consultations. If this option is not available or proves to be inadequate, it will be crucial to provide additional training for patients and health care professionals. It is therefore important to select a system that will not require time-consuming training, preferably one with an automated testing system (if available).

## Future Considerations

Looking toward the future, the population of heart patients in need of remote care is likely to grow given the prolongation of the COVID-19 pandemic. Using design thinking to improve telehealth for patients who are at risk of acute health problems, such as heart attack and atrial fibrillation, is therefore increasingly urgent. Remote diagnostic tools, such as remote electrocardiogram (ECG) technology, could be integrated into telehealth video communication software, as in smartphones [21]. Although there are some consumer devices on the market with up to six ECG leads [22,23], their use is still limited due to legal obstacles or financial concerns (linked to reimbursement).

## Limitations

This short design thinking study was limited due to the physical constraints of COVID-19. The data collection would have benefited from more field observations. In addition, to prevent delays in the implementation process of the video consultation software, the traditional problem exploration process started immediately through testing existing software solutions. Therefore, some problems and solutions for the stakeholders may remain unexplored.

## Conclusion

These initial insights highlight that even though financial regulations currently favor the use of video consultations [4,10,11], we identified many practical obstacles from a user perspective that have critical implications. Employing design thinking and involving all relevant stakeholders may help overcome these obstacles and aid further integration of telehealth and other medical device software. This further resonates with the call of the American Heart Association to conduct more human-centered research in this area [24]. In conclusion, employing design thinking to implement video consultations in cardiology and to further implement telehealth is crucial to build a resilient health care system that can address urgent needs beyond the COVID-19 pandemic.

## Abbreviations

COVID-19: coronavirus disease
ECG: electrocardiogram
eHealth: electronic health
GDPR: General Data Protection Regulation
ICD: implanted cardioverter-defibrillator device
IT: information technology
